# Impact of dietary live microbes and nondietary prebiotic/probiotic intake on osteoarthritis and rheumatoid arthritis development: Stratified findings from NHANES data

**DOI:** 10.1002/imo2.24

**Published:** 2024-08-17

**Authors:** Ang Liu, Qin Zhang, Xiao Liang, Zhujun Chao, Ruoran Zhou, Cheng Huang, Jun Lin

**Affiliations:** ^1^ Department of Orthopaedics, First Affiliated Hospital of Soochow University Soochow University Suzhou Jiangsu People's Republic of China; ^2^ Department of Orthopaedics, The Fourth Affiliated Hospital of Soochow University, Suzhou Dushu Lake Hospital Medical Center of Soochow University Suzhou Jiangsu People's Republic of China; ^3^ Department of Clinical Medicine, Suzhou Medical College Soochow University Suzhou China; ^4^ Department of Orthopaedics China‐Japan Friendship Hospital Beijing China

## Abstract

We selected participants from the NHANES database from 2005 to 2016 for this cross‐sectional analysis. Logistic regression and other analytical methods were utilized to analyze the relationship between the intake of dietary live microbes and nondietary prebiotic/probiotic and the prevalence of osteoarthritis (OA) and rheumatoid arthritis (RA). The findings demonstrated a direct relationship between the consumption of nondietary prebiotic/probiotic and the prevalence of developing OA, whereas a greater consumption of dietary live microbes is associated with a lower occurrence of RA. (Graphical abstract was created with BioRender.com).

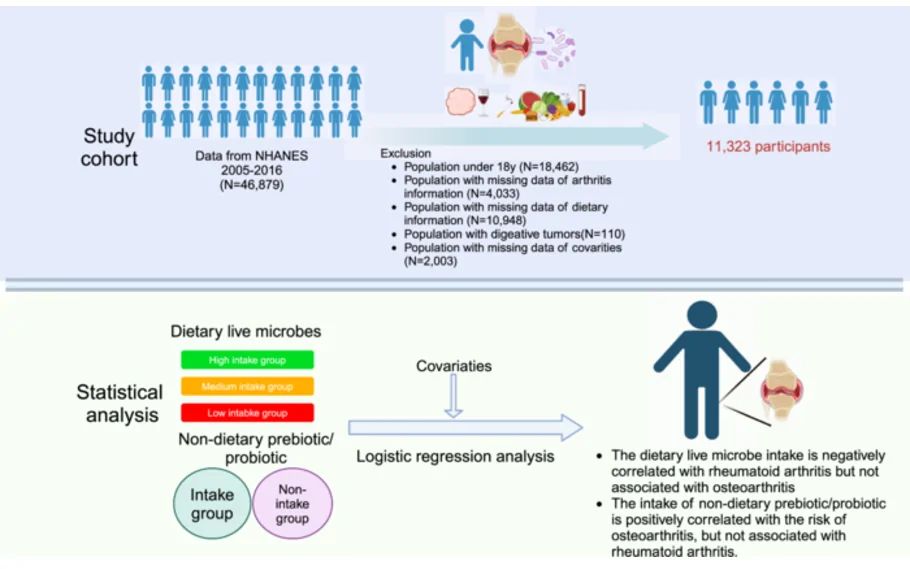

To the editor,

Osteoarthritis (OA) and rheumatoid arthritis (RA) are the most prevalent joint diseases globally, causing pain and disability. Scientific evidence has proven that joint diseases like OA and RA may arise and worsen as a result of altered gut microbiota. In terms of OA, disruption of the gut microbiota is considered to participate in the obesity‐related OA occurrence and progression [1]. Similarly, evidence has shown alterations in the gut, lung, and oral microbiota composition in RA patients, suggesting that there may be a link between RA and dysbiosis [2].

On the other hand, the potential for dietary and therapeutic intervention in OA and RA using the gut microbiota is gaining attention. Prebiotics are types of indigestible food components that specifically alter the composition or activity of the gut microbiota [3]. Probiotics are live, nonpathogenic microbes used to improve microbial balance and are regulated as dietary supplements and foods [4]. Studies have shown that a proper diet and prebiotic/probiotic supplementation can improve gut homeostasis [5]. Currently, research conducted on animals has demonstrated that probiotics have the ability to inhibit anterior cruciate ligament (ACL) rupture‐induced OA progression in mice [6], while relevant probiotic administration during the initial phases of RA can influence the onset of joint inflammation [7]. Nevertheless, more in‐depth cross‐sectional studies are required to find out the connection between consuming dietary live microbes and nondietary prebiotic/probiotic and OA and RA.

In summary, this study utilized data from the National Health and Nutrition Examination Survey (NHANES) to carry out a population‐based cross‐sectional analysis. The aim of this research was to clarify the relationship among dietary live microbes, nondietary prebiotic/probiotic, and the prevalence of OA and RA, and to assess whether this relationship is influenced by potential confounding factors.

## 1 RESULTS AND DISCUSSION

Through a cross‐sectional analysis of the NHANES database from 2005 to 2016, we preliminarily observed a potential link between higher dietary intake of live microbes and the reduced risk of RA and other types of arthritis, whereas the intake of nondietary prebiotic/probiotic demonstrated a positive connection with the risk of OA.

The research included 11,323 participants who were categorized into four groups. The weighted baseline participant characteristics of each group are displayed in Table S1. We can observe significant variations in statistics between the healthy control group and OA and RA patient groups (Table S1). In addition, Table S1 also presents the distribution of dietary intake of live microbes and nondietary prebiotic/probiotic within the groups of patients with OA and RA, as well as the group of healthy control. Specifically, individuals diagnosed with rheumatoid arthritis showed reduced consumption of dietary live microbes and nondietary prebiotic/probiotic, while OA patients exhibited an elevated intake of dietary live microbes and nondietary prebiotic/probiotic. Table S2 demonstrates the correlations between the different covariates and OA or RA. We can find that different covariates have different associations with the risk of OA and RA, some positively and some negatively (Table S2).

**Figure 1:**
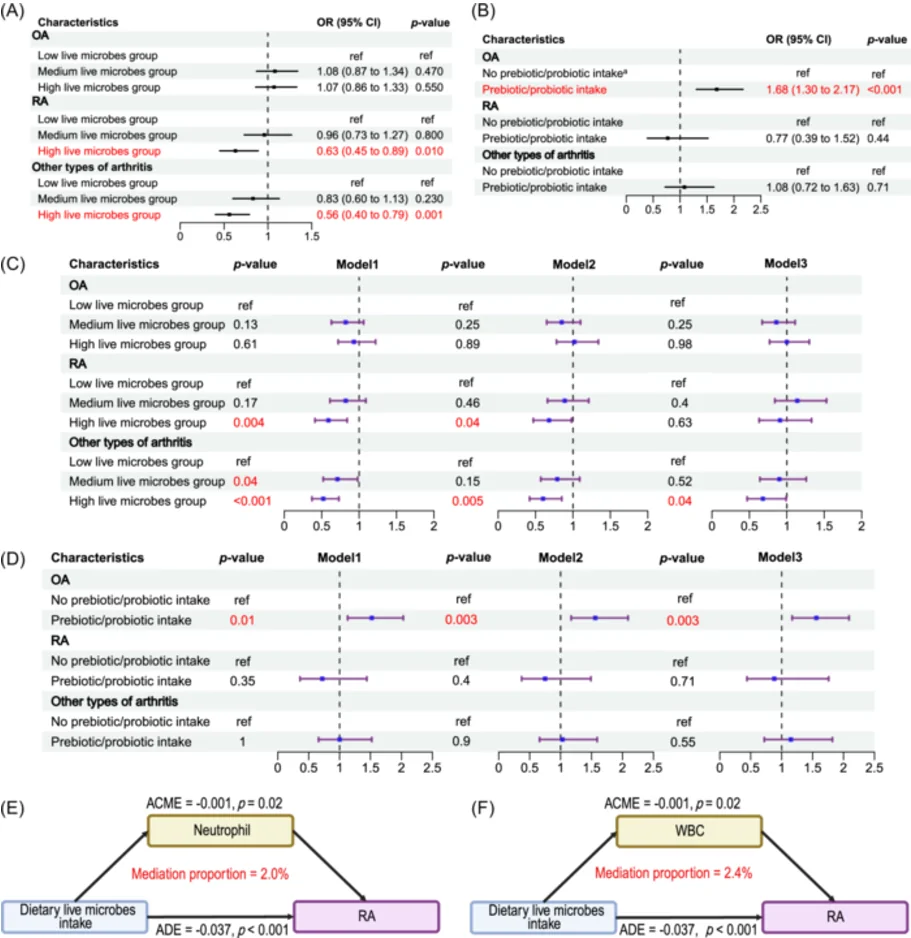
Impact of dietary live microbes and nondietary prebiotic/probiotic intake on osteoarthritis (OA) and rheumatoid arthritis (RA). (A) The univariate logistic regression analyses of the impact of dietary live microbes on the risk of OA and RA. (B) The univariate logistic regression analyses of the impact of nondietary prebiotic/probiotic intake on the risk of OA and RA. (C) The multivariable logistic regression analyses of the impact of dietary live microbes intake on the risk of OA and RA. (D) The multivariable logistic regression analyses of the impact of nondietary prebiotic/probiotic intake on the risk of OA and RA. (E) Mediation effects of neutrophil number in the associations of dietary live microbes intake and RA. (F) Mediation effects of WBC count in the associations of dietary live microbes intake and RA. a. Intake status refers to the intake of prebiotics or probiotics in either category or both; Model 1: adjusted for gender, age; Model 2: adjusted for gender, age, BMI, smoking, alcohol consumption; Model 3: Adjusted for age, gender, race/ethnicity, PIR, education level, marital status, BMI, smoking status, alcohol consumption, neutrophil number, WBC count and four dietary patterns; ACME refers to average causal mediation effects, which represent the indirect effect, while ADE stands for average direct effects.

The findings of this research indicate that the intake of dietary live microbes is not statistically significantly correlated with the prevalence of OA (Figure 1A,C). However, intake of nondietary prebiotic/probiotic is related to a significant rise in the prevalence of OA (Figure 1B,D). There is still controversy in the academic community about the association between intestinal microbiota and OA. To date, only one randomized controlled trial has demonstrated that the intake of *Lactobacillus casei* can improve the osteoarthritis index in patients [8]. Large‐scale clinical research data and comprehensive proof are still lacking to draw clear conclusions about the exact correlation between gut microbiota and susceptibility to OA. Additionally, the relationship between different bacterial species and the pathogenesis of OA requires further investigation. Although this study reveals an elevated risk of OA linked to the intake of nondietary prebiotic/probiotic, which differs significantly from some conclusions in existing literature, it further highlights the existence of mechanisms in the intricate interplay between gut microbiota and OA that we do not yet fully understand. To enhance our comprehension of this correlation, it is imperative to employ more sophisticated and systematic methods in research. This will involve clinical research with greater numbers of samples and exploration of the roles of different bacterial species in the development of OA, aiming to reveal the specific mechanisms underlying the interaction of prebiotic/probiotic with osteoarthritis and thus providing new strategies for OA's prevention and treatment.

The research showed that individuals who consumed more live microbes in their diet had a lower risk of developing rheumatoid arthritis and other nonosteoarthritis joint diseases (Figure 1A,C). Although the exact pathophysiological basis of RA remains to be elucidated, current research has established the pivotal significance of the gut microbiota in the development of RA. Research has demonstrated that the composition and functionality of the gut microbiota exhibit significant variability in RA patients [9]. Furthermore, evidence suggests a progressive decline in gut microbiota diversity as RA progresses [10]. Studies on diet and the live microbe have also demonstrated the significant role of various dietary patterns in shaping the composition of the intestinal microbiota [11]. For instance, the Mediterranean diet has been shown to improve RA through the modulation of gut microbiota and enhancement of intestinal barrier function [12]. Thus, an anti‐inflammatory diet containing live microbes has the potential to reduce the incidence of RA. These findings offer scientific data supporting the utilization of live microbe intake as a strategy to reduce the incidence of RA.

However, in this research, there was no statistically significant correlation observed between nondietary prebiotic/probiotic intake and a reduced risk of RA (Figure 1B,D). This result may be attributed to the dosage, type, individual difference, and bioavailability of nondietary prebiotic/probiotic supplements. Previous research has indicated that the effects of probiotics are strain‐specific [13]. Moreover, evidence suggests that not all types of *Lactobacillus* can alleviate arthritis symptoms [14]. Most of the related studies regarding the impact of probiotics on RA are currently based on animal models. Given the inability to fully replicate human disease pathologies in animal models, therapeutic effects in these models can only provide limited information [15]. Therefore, to precisely define the potential benefits of nondietary prebiotic/probiotic for rheumatoid arthritis patients, higher‐quality research with a larger sample size and longer durations of treatment are desperately required in this field. Meanwhile, future research should conduct comprehensive assessments to examine the precise impacts of prebiotic/probiotic components on the human gut microbiota and immune system, aiming to reveal the exact clinical benefits that such treatment approaches may offer.

Meanwhile, neutrophils and leukocytes have been shown to mediate the impacts of dietary live microbes on RA. The study found that different demographic characteristics and living habits can have an impact on neutrophil and white blood cell (WBC) counts (Table S3). The results of the univariate logistic regression showed an association between neutrophils, leukocytes, and RA, and the results of the multivariable logistic regression remained significant after the inclusion of some confounders (Tables S2 and S4). Univariate and multivariable linear regression results indicated that dietary live microbes were negatively associated with neutrophils and leukocytes (Tables S5–S6). Therefore, we used mediation analysis to investigate whether neutrophils and leukocytes mediate the modulation of RA by dietary live microbes. Based on the results of the above analyses, age, and gender were included as confounding factors in the mediation analysis. Mediation analyses showed that neutrophil number and WBC count mediated by 2.0% and 2.4%, respectively (Figure 1E,F). Relevant research has demonstrated that the microbiota has an influence on modulating the immune system of the host [16]. Leukocytes and neutrophils have also been demonstrated to have a significant impact on the pathogenic course of RA [17, 18]. Our mediation analyses of the association between gut microbiota and RA further validated these ideas.

This study also further analyzed in several subgroups to investigate those demographic characteristics such as alcohol consumption, marital status, body mass index (BMI), different dietary patterns, neutrophils, and leukocytes may influence the relevance between the intake of dietary live microbes and nondietary prebiotic/probiotic and the risk of arthritis (Figure S2). These views are supported by epidemiological and pathogenic studies on RA [19, 20].

In the sensitivity analysis, given the high number of missing values for intake of nondietary prebiotic/probiotic (*N* = 10,947), accounting for more than 40% of the sample, this study further conducted an analysis excluding this variable to investigate the connection between dietary live microbes intake and RA as well as other types of arthritis. The logistic regression analysis results showed that the relationship between dietary consumption of live microbes and RA and other types of arthritis remained consistent with previous findings after excluding the variable with missing values (Tables S7–S8). The evidence indicated a positive correlation between consuming dietary live microbes and a lower risk of RA and other types of arthritis.

It is necessary to take into consideration several limitations when assessing the findings of this research. First, as a result of this study's cross‐sectional design, the observed correlations between the consumption of dietary live microbes and nondietary prebiotic/probiotic and the symptoms of arthritis cannot be directly inferred as causal relationships. Second, this study cannot distinguish between various types of arthritis during the statistical classification process, which limited our correlation analyses to other specific types of arthritis in addition to RA and OA. Additionally, despite adjusting for multiple major covariates during the analysis, there may still be residual confounding factors that were not included in the model design or could not be adjusted for due to missing data. In this study, regarding the assessment of dietary live microbes consumption and the severity of joint inflammation, we could only consider intake levels and prevalence due to the limitations of the database. While this classification method of microbes and prebiotic/probiotic have advantages in terms of efficiency and generalizability, it may still result in a certain degree of measurement error compared to direct measurement of content. Because the database has no assessment of arthritis‐related scores and severity indicators, we were unable to further explore the relationship between intake and severity of joint inflammation. These issues deserve to be considered in further future experiments. Furthermore, the dietary supplement data used in this study were based on intake records from the past 30 days and did not reflect the duration of use of prebiotic or probiotic supplements, limiting our ability to differentiate between short‐term and long‐term intake effects. Lastly, considering that the study data were derived from NHANES in the United States, whose sample represents the US population, it should be cautious when generalizing the study results to other countries and populations.

Future research should include prospective cohort studies and randomized controlled trials to investigate the causal relationship between the consumption of dietary live microbes, nondietary prebiotic/probiotic, and the risk of OA and RA. Furthermore, researchers should focus on the impact of different types and doses of prebiotic/probiotic on OA and RA, as well as their interactions with participants' genetic backgrounds and lifestyles. In addition, future studies should involve differences in the distinct pathways via which the gut microbiota influences osteoarthritis and rheumatoid arthritis.

## 2 CONCLUSION

To summarize, the study indicated that the risk of RA and other non‐OA types of arthritis are negatively correlated with dietary live microbe intake. However, OA is not significantly associated with dietary live microbe consumption. Conversely, the intake of nondietary prebiotic/probiotic is positively correlated with the risk of OA but does not appear to be significantly associated with other non‐OA types of arthritis. Taking into account the constraints of current research, future studies should use more rigorous design methods, incorporate gut microbiota and metabolomic analyses to better understand complex interactions between gut microbiota and arthritis, and explore personalized nutritional intervention strategies.

## AUTHOR CONTRIBUTIONS

**Ang Liu**: Conceptualization; methodology; formal analysis; data Curation; writing—original draft. **Qin Zhang**: Methodology; formal analysis; and investigation. **Xiao Liang**: Methodology; formal analysis; and investigation. **Zhujun Chao**: Investigation; validation. **Ruoran Zhou**: Investigation; validation. **Cheng Huang**: Writing—review and editing; supervision; project administration; funding acquisition. **Jun Lin**: Conceptualization; writing—review and editing; supervision; project administration; funding acquisition.

## CONFLICT OF INTEREST STATEMENT

The authors declare no conflict of interest.

## ETHICS STATEMENT

The NHANES project was conducted with the approval of the Research Ethics Review Board of the National Center for Health Statistics and required all participants to sign an informed consent form; minor participants (under 18 years of age) required the consent of a parent or legal guardian. The NHANES data set used in this study was publicly available and no additional authorization or ethical approval was required.

## Supporting information


**Figure S1:** Flow chart for participants selection.
**Figure S2:** The forest scatter of subgroup analysis.
**Table S1:** Demographic information of the participants in the research.
**Table S2:** Univariate logistic regression analysis between covariates and different types of arthritis.
**Table S3:** Univariate linear regression analysis between covariates and neutrophil number and WBC count.
**Table S4:** Multivariable logistic regression analysis between neutrophil number and WBC count and RA.
**Table S5:** Univariate linear regression analyses between dietary live microbes and neutrophil number and WBC count.
**Table S6:** Multivariable linear regression analyses between dietary live microbes and neutrophil number and WBC count.
**Table S7:** Univariate logistic regression analysis between dietary live microbes and RA and other non‐OA arthritis after removing non‐dietary prebiotic/probiotic data.
**Table S8:** Multivariable logistic regression analysis between dietary live microbes and RA and other non‐OA arthritis after removing non‐dietary prebiotic/probiotic data.

## Data Availability

All raw data in this work was available at https://www.cdc.gov/nchs/nhanes/index.htm and https://github.com/LiuAngLA/microbe-prebiotic-probiotic-and-arthritis-NHANES. Supporting Information (methods, figures, tables, scripts, graphical abstract, slides, videos, Chinese translated version, and update materials) may be found in the online DOI or iMeta Science https://www.imeta.science/imetaomics/.
