# Supplementary material for: Impact of dietary live microbes and nondietary prebiotic/probiotic intake on osteoarthritis and rheumatoid arthritis development: Stratified findings from NHANES data
Source: IMetaOmics. 2024 Aug 17;1(1):e24. doi: 10.1002/imo2.24 (PMC12806318; doi:10.1002/imo2.24)
Supplement: Supplementary file 1 — Figure S1: Flow chart for participants selection. Figure S2: The forest scatter of subgroup analysis. Table S1: Demographic information of the participants in the research. Table S2: Univariate logistic regression analysis between covariates and different types of arthritis. Table S3: Univariate linear regression analysis between covariates and neutrophil number and WBC count. Table S4: Multivariable logistic regression analysis between neutrophil number and WBC count and RA. Table S5: Univariate linear regression analyses between dietary live microbes and neutrophil number and WBC count. Table S6: Multivariable linear regression analyses between dietary live microbes and neutrophil number and WBC count. Table S7: Univariate logistic regression analysis between dietary live microbes and RA and other non‐OA arthritis after removing non‐dietary prebiotic/probiotic data. Table S8: Multivariable logistic regression analysis between dietary live microbes and RA and other non‐OA arthritis after removing non‐dietary prebiotic/probiotic data. [file IMO2-1-e24-s001.docx]

**Supporting information** to “Impact of Dietary Live Microbes and Non-Dietary Prebiotic/Probiotic Intake on Osteoarthritis and Rheumatoid Arthritis Development: Stratified Findings from NHANES Data”

**Supporting information includes:**

Methods

Supplementary Figure S1-2:

Figure S1 Flow chart for participants selection.

Figure S2. The forest scatter of subgroup analysis.

Supplementary Table S1-8:

Table S1. Demographic information of the participants in the research.

Table S2. Univariate logistic regression analysis between covariates and different types of arthritis.

Table S3. Univariate linear regression analysis between covariates and neutrophil number and WBC count.

Table S4. Multivariable logistic regression analysis between neutrophil number and WBC count and RA.

Table S5. Univariate linear regression analyses between dietary live microbes and neutrophil number and WBC count.

Table S6. Multivariable linear regression analyses between dietary live microbes and neutrophil number and WBC count.

Table S7. Univariate logistic regression analysis between dietary live microbes and RA and other non-OA arthritis after removing non-dietary prebiotic/probiotic data.

Table S8. Multivariable logistic regression analysis between dietary live microbes and RA and other non-OA arthritis after removing non-dietary prebiotic/probiotic data.

**Methods**

Data source and study population

In this work, we employed data from eight survey cycles of NHANES conducted between 2005 and 2016 for a cross-sectional analysis. From a total of 46,879 participants with complete weight information, we initially excluded 18,462 underage individuals. Next, 4,033 participants were further excluded due to missing arthritis-related data; another 10,948 participants were excluded due to incomplete dietary data; another 110 participants were excluded due to digestive tumors and finally, a further 2,003 participants were excluded due to missing information on covariates. Consequently, 11,323 patients in all were enrolled in this study. Figure S1 depicts the comprehensive selection procedure.


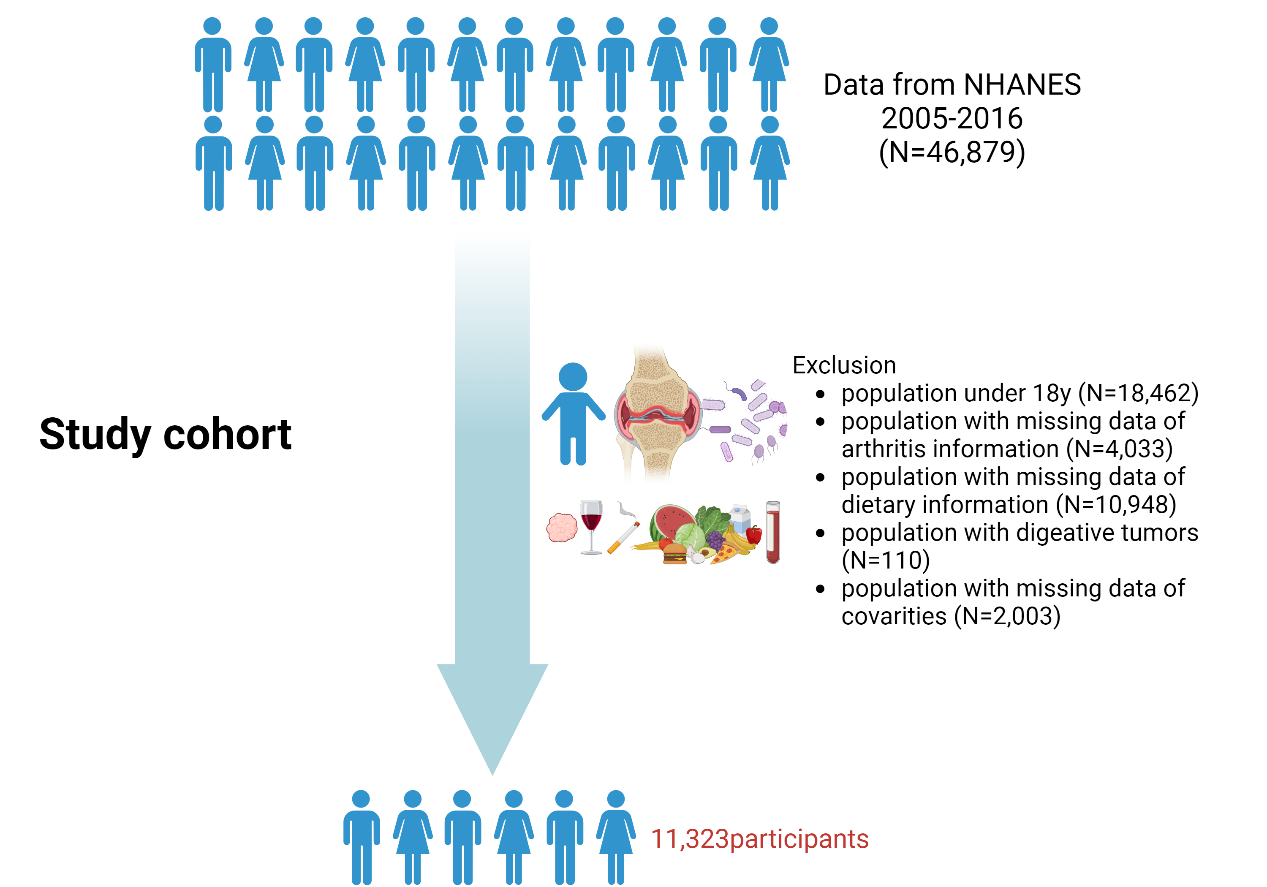


**Figure S1.** Flow chart for participants selection. (Created with BioRender.com)

Assessment of arthritis

In this study, the participants' arthritis status was assessed based on self-reported information obtained during in-person interviews. All participants older than eighteen years were asked a key question to determine their arthritis diagnosis: " Has a doctor or other health professional ever told {you/SP} that {you/s/he} . . .had arthritis?" For those participants who answered yes, further inquiries were made regarding the specific type of arthritis: "Which type of arthritis was it?" Participants were classified into four groups based on their answers to the two questions: healthy control group, OA patient group, RA patient group, and other types of arthritis patient group.

Dietary live microbe intake

Dietary intake data were collected through standardized in-person interviews for a 24-hour dietary recall conducted by professionally trained investigators. These data were then matched with the Food and Nutrient Database specifically developed for the NHANES cycles by the United States Department of Agriculture (USDA) to ensure the accuracy and reliability of the dietary data. Based on this, we adopted the approach used by Marco et al. to categorize the consumption of live microbes in the diet[1]. Marco and colleagues identified 9,388 unique food codes within the NHANES database, representing 48 different categories of food. To accurately capture the expected variability of live microbes in different food categories, we classified them into three levels based on the number of live microbes present in the food: low (<10^4^ CFU/gram), medium (10^4^-10^7^ CFU/gram), and high (>10^7^ CFU/gram). The "low" level refers to foods that had undergone pasteurization with a live microbe count lower than 10^4^ CFU/gram, the "medium" level includes unpeeled fresh fruits and vegetables with live microbe counts ranging from 10^4^ to 10^7^ CFU/gram, and the "high" level refers to unpasteurized fermented foods and supplements containing more than 10^7^ CFU/gram of live microbes. According to this classification system, participants' dietary live microbes intake was classified into three groups based on a classification system: those with low intake (including participants who only consume foods with a low level of live microbes), those with medium intake (including participants who consume foods with a medium level but not a high level of live microbes), and those with high intake (including participants who consume foods with a high level of live microbes). This grouping method is consistent with existing relevant literature[2-4].

Non-dietary prebiotic/probiotic intake

At the present time, the Food and Drug Administration (FDA) in the United States does not directly regulate probiotic products, which means that these products can only be sold in the market as dietary supplements. O'Connor et al. have compiled a list of prebiotic and probiotic names in the literature published in 2021, providing an important reference for identifying specific prebiotic and probiotic species[5]. In addition, lactulose is also a prebiotic and is regulated by the FDA. In our study, a 30-day dietary supplement questionnaire was utilized to obtain data about participants' consumption of prebiotic and probiotic dietary supplements in the 30 days leading up to the interview to assess their intake levels.

Covariates

When constructing our research model, we referred to the existing literature, which involves studies on the relevance of dietary live microbes, non-dietary prebiotics/probiotics, and arthritis. Based on these studies, we carefully selected and evaluated a range of covariates aimed at controlling for confounding factors that could affect the associations between arthritis inflammation and intake of dietary live microbes. These covariates included, but were not limited to: age, gender, race/ethnicity (Mexican-American, non-Hispanic Black, non-Hispanic White, or other race), poverty income ratio (PIR), education level, marital status, body mass index (BMI), smoking status, drinking habits, and dietary patterns. Education level was categorized as under high school, high school or equivalent, or higher than high school. Marital status was categorized as married or cohabiting, divorced or separated or widowed, or never married. PIR was classified into three levels: low (<1.31), medium (1.31-3.5), and high (>3.5). BMI was a categorical variable calculated based on recorded height and weight from medical examination data (BMI = weight (kg) / height (m)^2^), categorized according to internationally recognized thresholds as underweight/normal weight (<25 kg/m^2^), overweight (25≤BMI≤29.9 kg/m^2^), or obese (>29.9 kg/m^2^). Smoking status was a categorical variable, classified as "smokers" or "non-smokers" based on whether individuals were asked if they had smoked over 100 cigarettes in their lifetime. Alcohol information was categorized as "drinkers" or "non-drinkers" based on whether individuals were asked if they consumed more than 12 drinks per year. Based on previous studies, four relevant dietary models were included in the study, namely Healthy Eating Index 2020(HEI), Alternative Healthy Eating Index (AHEI), Alternate Mediterranean Diet Score (AMED), and Dietary Inflammation Index (DII)[6-8]. The Dietary Index for Gut Microbiota was excluded due to a lack of validation in relevant studies[9]. Quintiles of their dietary scores were included in the statistical analysis: Q1(lowest), Q2, Q3(typical), Q4, Q5(highest)[6, 10, 11]. Neutrophil number and white blood cell count were measured at the NHANES Mobile Examination Centers (MECs). Quintiles of them were included in the statistical analysis: Q1(lowest), Q2, Q3(typical), Q4, Q5(highest). Detailed laboratory methods are available on the NHANES website.

Statistical analysis

After strict screening based on inclusion and exclusion criteria, the final study cohort was identified from the NHANES database. Detailed data cleaning was performed for the exposure variables, outcome variables, and covariates in the database to guarantee the precision of the analysis. All data analyses were performed following the analytical guidelines provided by NHANES.

Descriptive statistical analysis was further performed in this study to summarize the demographic characteristics and anthropometric data of the study population. During statistical analysis, we strictly adhered to NHANES stratified complex sampling design and applied appropriate sample weighting methods to guarantee the accuracy and dependability of the results^3^. For continuous variables, the descriptive data were shown using the mean (standard error). The frequencies and matching percentages were used to express the categorical variables. To analyze disparities among distinct groups, statistical tests suitable for different data types and distributions were employed. Specifically, for continuous variables, analysis of variance (ANOVA) was utilized to evaluate the importance of mean differences among different groups. The chi-square test was employed for the assessment of statistical distinctions in frequency distributions among groups for categorical variables.

To explore the potential associations between intake of dietary live microbes as well as non-dietary prebiotics/probiotics and the risk of OA and RA, this study conducted univariate and multivariate logistic regression analyses. In the multivariable models, adjustment was made for several key confounding variables, including but not limited to age, gender, and BMI. To comprehend the interactions between each covariate and OA and RA more deeply, subgroup analyses were performed, and heterogeneity among subgroups was assessed. This heterogeneity was evaluated using multivariable logistic regression models, and the interaction of subgroups with OA and RA was tested by likelihood ratio tests. We performed linear regression analyses to explore the association between the level of intake of dietary live microbes and neutrophils and leukocytes. Mediation analyses were conducted to explore direct and indirect relationships and the magnitude of the mediating effect. These analyses helped to further elucidate the relationship between these factors and the risk of different types of arthritis.

R (version 4.2.2) statistical software was utilized for the statistical analyses. Statistical significance in this study was determined using a significance level of P < 0.05.

**References**

1. Marco, Maria L., Robert Hutkins, Colin Hill, Victor L. Fulgoni, Christopher J. Cifelli, Jaime Gahche, Joanne L. Slavin, Daniel Merenstein, Daniel J. Tancredi, Mary E. Sanders. 2022. “A Classification System for Defining and Estimating Dietary Intake of Live Microbes in US Adults and Children.” *The Journal of Nutrition* 152: 1729-1736. <https://doi.org/10.1093/jn/nxac074>

2. Wang, Xuefei, Huaicheng Wang, Qianwen Yu, Shibo Fu, Zeqiang Yang, Qinyong Ye, Fabin Lin, Guoen Cai. 2024. “"High dietary live microbe intake is correlated with reduced risk of depressive symptoms: A cross-sectional study of NHANES 2007-2016".” *Journal of Affective Disorders* 344: 198-206. <https://doi.org/10.1016/j.jad.2023.10.015>

3. Tang, Haoxian, Xuan Zhang, Nan Luo, Jingtao Huang, Yanqiao Zhu. 2024. “Association of Dietary Live Microbes and Nondietary Prebiotic/Probiotic Intake With Cognitive Function in Older Adults: Evidence From NHANES.” *The Journals of Gerontology: Series A* 79: glad175. <https://doi.org/10.1093/gerona/glad175>

4. Han, Lu, Qi Wang. 2022. “Association of Dietary Live Microbe Intake with Cardiovascular Disease in US Adults: A Cross-Sectional Study of NHANES 2007-2018.” *Nutrients* 14: 4908. <https://doi.org/10.3390/nu14224908>

5. O'Connor, Lauren E., Jaime J. Gahche, Kirsten A. Herrick, Cindy D. Davis, Nancy Potischman, Ashley J. Vargas. 2021. “Nonfood Prebiotic, Probiotic, and Synbiotic Use Has Increased in US Adults and Children From 1999 to 2018.” *Gastroenterology* 161: 476-486.e473. <https://doi.org/10.1053/j.gastro.2021.04.037>

6. Wang, Peilu, Mingyang Song, A. Heather Eliassen, Molin Wang, Teresa T. Fung, Steven K. Clinton, Eric B. Rimm, et al. 2023. “Optimal dietary patterns for prevention of chronic disease.” *Nature Medicine* 29: 719-728. <https://doi.org/10.1038/s41591-023-02235-5>

7. Petersson, Sara, Elena Philippou, Carrie Rodomar, Elena Nikiphorou. 2018. “The Mediterranean diet, fish oil supplements and Rheumatoid arthritis outcomes: evidence from clinical trials.” *Autoimmunity Reviews* 17: 1105-1114. <https://doi.org/10.1016/j.autrev.2018.06.007>

8. Jandari, Sajedeh, Negin Mosalmanzadeh, Mohammad Reza Shadmand Foumani Moghadam, Davood Soleimani, Nitin Shivappa, James R. Hébert, Mohammadhassan Jokar, et al. 2021. “Dietary inflammatory index and healthy eating index-2015 are associated with rheumatoid arthritis.” *Public Health Nutrition* 24: 6007-6014. <https://doi.org/10.1017/S1368980021001105>

9. Kase, Bezawit E., Angela D. Liese, Jiajia Zhang, Elizabeth Angela Murphy, Longgang Zhao, Susan E. Steck. 2024. “The Development and Evaluation of a Literature-Based Dietary Index for Gut Microbiota.” *Nutrients* 16: 1045. <https://doi.org/10.3390/nu16071045>

10. Shan, Zhilei, Fenglei Wang, Yanping Li, Megu Y. Baden, Shilpa N. Bhupathiraju, Dong D. Wang, Qi Sun, et al. 2023. “Healthy Eating Patterns and Risk of Total and Cause-Specific Mortality.” *JAMA internal medicine* 183: 142-153. <https://doi.org/10.1001/jamainternmed.2022.6117>

11. Fadnes, Lars T., Carlos Celis-Morales, Jan-Magnus Økland, Solange Parra-Soto, Katherine M. Livingstone, Frederick K. Ho, Jill P. Pell, et al. 2023. “Life expectancy can increase by up to 10 years following sustained shifts towards healthier diets in the United Kingdom.” *Nature Food* 4: 961-965. <https://doi.org/10.1038/s43016-023-00868-w>


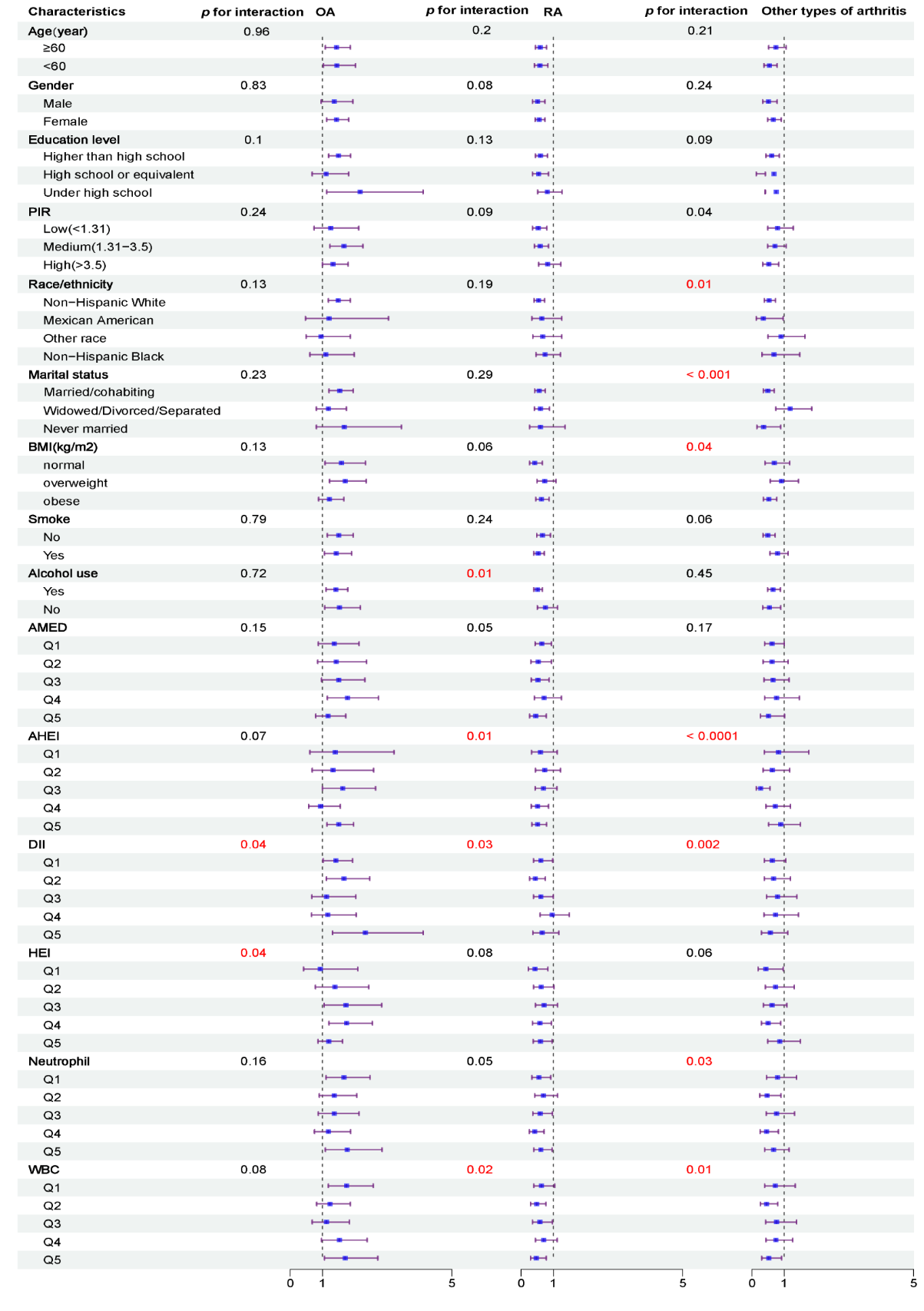


**Figure S2. The forest scatter of subgroup analysis**

Notes: Univariate logistic regression analyses and interaction tests were used in the subgroup analyses.

**Table S1. Demographic information of the participants in the research**

| **Characteristics** | **Healthy (N=8397)** | **OA**  **(N=1724)** | **RA**  **(N=712)** | **Other types of arthritis (N=490)** | ***P*** |
| --- | --- | --- | --- | --- | --- |
| Age (years) |  |  |  |  | < 0.0001 |
| Mean(SE) | 45.99(0.32) | 63.28(0.45) | 59.56(0.65) | 55.98(0.98) |  |
| Gender (%) |  |  |  |  | < 0.0001 |
| Female | 4473(52.46) | 1165(68.30) | 431(61.81) | 288(61.46) |  |
| Male | 3924(47.54) | 559(31.70) | 281(38.19) | 202(38.54) |  |
| Education level (%) |  |  |  |  | < 0.0001 |
| Higher than high school | 5415(71.37) | 1075(67.17) | 340(52.40) | 252(57.10) |  |
| High school or equivalent | 1704(19.55) | 374(20.95) | 179(26.60) | 133(28.20) |  |
| Under high school | 1278(9.08) | 275(11.88) | 193(21.00) | 105(14.70) |  |
| Marital status (%) |  |  |  |  | < 0.0001 |
| Married/cohabiting | 5472(67.73) | 1025(64.38) | 400(63.58) | 303(63.14) |  |
| Divorced/separated/widowed | 1544(15.91) | 586(29.75) | 258(29.99) | 135(27.03) |  |
| Never married | 1381(16.36) | 113(5.87) | 54(6.43) | 52(9.83) |  |
| Race/ethnicity (%) |  |  |  |  | < 0.0001 |
| Mexican-American | 1126(6.54) | 105(2.47) | 90(6.27) | 40(3.00) |  |
| Non-Hispanic Black | 1382(7.51) | 239(5.63) | 183(12.58) | 79(7.20) |  |
| Non-Hispanic White | 4275(74.26) | 1192(85.80) | 354(74.40) | 293(80.56) |  |
| Other race | 1614(11.69) | 188(6.11) | 85(6.74) | 78(9.23) |  |
| PIR (%) |  |  |  |  | < 0.0001 |
| Low(<1.31) | 1879(14.57) | 369(12.86) | 251(25.94) | 133(20.79) |  |
| Medium(1.31-3.5) | 3091(32.60) | 684(36.49) | 282(41.27) | 215(43.91) |  |
| High(>3.5) | 3427(52.82) | 671(50.66) | 179(32.79) | 142(35.31) |  |
| BMI (kg/m^2^) (%) |  |  |  |  | < 0.0001 |
| Underweight/normal (<25) | 2710(33.95) | 374(23.15) | 163(26.50) | 99(19.17) |  |
| Overweight (25-29.9) | 2939(35.21) | 547(32.94) | 202(25.45) | 145(28.23) |  |
| Obese (>29.9) | 2748(30.84) | 803(43.91) | 347(48.05) | 246(52.60) |  |
| Smoke (%) |  |  |  |  | < 0.0001 |
| No | 4923(58.37) | 845(49.44) | 325(43.15) | 217(41.50) |  |
| Yes | 3474(41.63) | 879(50.56) | 387(56.85) | 273(58.50) |  |
| Alcohol use (%) |  |  |  |  | < 0.0001 |
| No | 2209(20.55) | 530(26.37) | 231(29.71) | 149(26.49) |  |
| Yes | 6188(79.45) | 1194(73.63) | 481(70.29) | 341(73.51) |  |
| Neutrophil (1000 cells/ul) |  |  |  |  | 0.001 |
| Mean(SE) | 4.28(0.03) | 4.36(0.06) | 4.63(0.11) | 4.55(0.12) |  |
| WBC (1000 cells/ul) |  |  |  |  | 0.03 |
| Mean(SE) | 7.21(0.04) | 7.25(0.08) | 7.55(0.17) | 7.51(0.15) |  |
| AMED(%) |  |  |  |  | 0.13 |
| Q1 | 2347(28.62) | 426(25.07) | 233(33.37) | 144(31.39) |  |
| Q2 | 1490(17.05) | 286(17.52) | 120(15.45) | 97(19.59) |  |
| Q3 | 1560(19.13) | 331(18.05) | 122(18.83) | 100(21.20) |  |
| Q4 | 1350(15.70) | 285(16.87) | 112(16.76) | 66(12.33) |  |
| Q5 | 1650(19.50) | 396(22.49) | 125(15.59) | 83(15.49) |  |
| AHEI(%) |  |  |  |  | < 0.001 |
| Q1 | 1083(12.05) | 172(9.75) | 96(14.72) | 58(14.21) |  |
| Q2 | 1267(14.68) | 205(11.32) | 130(19.18) | 98(19.87) |  |
| Q3 | 1509(17.24) | 310(16.75) | 132(15.59) | 93(19.64) |  |
| Q4 | 1725(20.01) | 376(21.36) | 171(22.20) | 99(20.38) |  |
| Q5 | 2813(36.02) | 661(40.81) | 183(28.31) | 142(25.91) |  |
| DII(%) |  |  |  |  | < 0.0001 |
| Q1 | 2663(35.06) | 510(30.94) | 159(24.35) | 122(27.91) |  |
| Q2 | 1924(22.89) | 409(23.76) | 144(21.40) | 94(19.72) |  |
| Q3 | 1568(18.00) | 255(14.16) | 146(19.75) | 112(24.30) |  |
| Q4 | 1273(14.31) | 285(17.90) | 134(19.93) | 79(15.59) |  |
| Q5 | 969(9.75) | 265(13.25) | 129(14.57) | 83(12.48) |  |
| HEI(%) |  |  |  |  | 0.01 |
| Q1 | 1240(14.94) | 194(12.20) | 113(15.02) | 73(13.08) |  |
| Q2 | 1395(16.12) | 257(15.15) | 130(18.76) | 88(19.58) |  |
| Q3 | 1548(18.99) | 300(17.72) | 152(23.44) | 100(26.08) |  |
| Q4 | 1758(20.79) | 374(21.47) | 143(17.53) | 107(18.58) |  |
| Q5 | 2456(29.16) | 599(33.46) | 174(25.25) | 122(22.68) |  |
| Dietary live microbe (%) |  |  |  |  | 0.01 |
| Low | 2608(28.22) | 468(26.74) | 264(32.39) | 181(35.62) |  |
| Medium | 3562(40.65) | 771(41.72) | 321(45.01) | 214(42.39) |  |
| High | 2227(31.13) | 485(31.54) | 127(22.60) | 95(21.98) |  |
| Prebiotic/probiotic^a^ (%) |  |  |  |  | <0.001 |
| No | 7908(92.91) | 1583(88.64) | 684(94.47) | 460(92.38) |  |
| Yes | 489(7.09) | 141(11.36) | 28(5.53) | 30(7.62) |  |

Notes: ^a^Intake status refers to the intake of prebiotics or probiotics in either category or both; ANOVA was performed to evaluate mean differences between groups for continuous variables; The chi-square test was employed to analyze differences in frequency distributions among groups for categorical variables.

**Table S2.** **Univariate logistic regression analysis between covariates and different types of arthritis**

| **Characteristics** | **OA** | | **RA** | | **Other types of arthritis** | |
| --- | --- | --- | --- | --- | --- | --- |
|  | **OR (95%CI)** | ***P*** | **OR (95%CI)** | ***P*** | **OR (95%CI)** | ***P*** |
| Age | 1.08(1.07,1.08) | <0.0001 | 1.05(1.05,1.06) | <0.0001 | 1.04(1.03,1.05) | <0.0001 |
| Gender |  |  |  |  |  |  |
| Female | ref | ref | ref | ref | ref | ref |
| Male | 0.51(0.43,0.61) | <0.0001 | 0.68(0.52,0.89) | 0.005 | 0.69(0.53,0.91) | 0.01 |
| Education level |  |  |  |  |  |  |
| Higher than high school | ref | ref | ref | ref | ref | ref |
| High school or equivalent | 1.14(0.95,1.37) | 0.16 | 1.85(1.36,2.52) | <0.001 | 1.80(1.28,2.55) | 0.001 |
| Under high school | 1.39(1.13,1.71) | 0.002 | 3.15(2.27,4.37) | <0.0001 | 2.02(1.56,2.62) | <0.0001 |
| Marital status |  |  |  |  |  |  |
| Married/cohabiting | ref | ref | ref | ref | ref | ref |
| Divorced/separated/widowed | 1.97(1.62,2.38) | <0.0001 | 2.01(1.60,2.52) | <0.0001 | 1.82(1.36,2.44) | <0.0001 |
| Never married | 0.38(0.29,0.50) | <0.0001 | 0.42(0.25,0.71) | 0.001 | 0.64(0.40,1.03) | 0.07 |
| Race/ethnicity |  |  |  |  |  |  |
| Mexican-American | ref | ref | ref | ref | ref | ref |
| Non-Hispanic Black | 1.99(1.46,2.71) | <0.0001 | 1.75(1.23,2.48) | 0.002 | 2.09(1.11,3.92) | 0.02 |
| Non-Hispanic White | 3.06(2.31,4.06) | <0.0001 | 1.05(0.77,1.41) | 0.77 | 2.36(1.34,4.18) | 0.004 |
| Other race | 1.39(0.97,1.98) | 0.07 | 0.60(0.39,0.92) | 0.02 | 1.72(0.88,3.36) | 0.11 |
| PIR |  |  |  |  |  |  |
| Low (<1.31) | ref | ref | ref | ref | ref | ref |
| Medium (1.31-3.5) | 1.27(1.01,1.59) | 0.04 | 0.71(0.52,0.97) | 0.03 | 0.94(0.68,1.32) | 0.73 |
| High (>3.5) | 1.09(0.88,1.35) | 0.44 | 0.35(0.25,0.48) | <0.0001 | 0.47(0.32,0.68) | <0.0001 |
| BMI (kg/m2) |  |  |  |  |  |  |
| Underweight/normal (<25) | ref | ref | ref | ref | ref | ref |
| Overweight (25-29.9) | 1.37(1.10,1.71) | 0.01 | 0.93(0.66,1.30) | 0.65 | 1.42(1.01,2.00) | 0.04 |
| Obese (>29.9) | 2.09(1.72,2.53) | <0.0001 | 2.00(1.45,2.76) | <0.0001 | 3.02(2.11,4.33) | <0.0001 |
| Smoke |  |  |  |  |  |  |
| No | ref | ref | ref | ref | ref | ref |
| Yes | 1.43(1.24,1.65) | <0.0001 | 1.85(1.44,2.37) | <0.0001 | 1.98(1.53,2.56) | <0.0001 |
| Alcohol use |  |  |  |  |  |  |
| No | ref | ref | ref | ref | ref | ref |
| Yes | 0.72(0.60,0.87) | <0.001 | 0.61(0.47,0.80) | <0.001 | 0.72(0.53,0.98) | 0.04 |
| Neutrophil | 1.03(0.99,1.07) | 0.16 | 1.12(1.05,1.19) | <0.001 | 1.09(1.01,1.18) | 0.02 |
| WBC | 1.01(0.98,1.04) | 0.59 | 1.05(1.01,1.10) | 0.01 | 1.05(1.00,1.09) | 0.03 |
| AMED |  |  |  |  |  |  |
| Q1 | ref | ref | ref | ref | ref | ref |
| Q2 | 1.17(0.92,1.49) | 0.19 | 0.78(0.55,1.09) | 0.15 | 1.05(0.69,1.59) | 0.83 |
| Q3 | 1.08(0.88,1.33) | 0.48 | 0.84(0.59,1.21) | 0.35 | 1.01(0.69,1.47) | 0.96 |
| Q4 | 1.23(0.94,1.60) | 0.13 | 0.92(0.65,1.30) | 0.62 | 0.72(0.47,1.08) | 0.11 |
| Q5 | 1.32(1.05,1.65) | 0.02 | 0.69(0.43,1.10) | 0.11 | 0.72(0.46,1.14) | 0.16 |
| AHEI |  |  |  |  |  |  |
| Q1 | ref | ref | ref | ref | ref | ref |
| Q2 | 0.95(0.72,1.25) | 0.73 | 1.07(0.69,1.66) | 0.76 | 1.15(0.71,1.86) | 0.57 |
| Q3 | 1.20(0.88,1.64) | 0.25 | 0.74(0.48,1.15) | 0.18 | 0.97(0.61,1.52) | 0.88 |
| Q4 | 1.32(1.00,1.74) | 0.05 | 0.91(0.62,1.34) | 0.62 | 0.86(0.55,1.35) | 0.52 |
| Q5 | 1.40(1.07,1.84) | 0.02 | 0.64(0.43,0.97) | 0.04 | 0.61(0.37,1.00) | 0.05 |
| DII |  |  |  |  |  |  |
| Q1 | ref | ref | ref | ref | ref | ref |
| Q2 | 1.18(0.96,1.44) | 0.11 | 1.35(0.97,1.87) | 0.07 | 1.08(0.79,1.49) | 0.63 |
| Q3 | 0.89(0.72,1.10) | 0.29 | 1.58(1.06,2.36) | 0.03 | 1.70(1.09,2.63) | 0.02 |
| Q4 | 1.42(1.03,1.94) | 0.03 | 2.01(1.41,2.85) | <0.001 | 1.37(0.89,2.11) | 0.15 |
| Q5 | 1.54(1.21,1.97) | <0.001 | 2.15(1.46,3.18) | <0.001 | 1.61(1.05,2.47) | 0.03 |
| HEI |  |  |  |  |  |  |
| Q1 | ref | ref | ref | ref | ref | ref |
| Q2 | 1.15(0.84,1.58) | 0.38 | 1.16(0.75,1.78) | 0.5 | 1.39(0.84,2.31) | 0.2 |
| Q3 | 1.14(0.88,1.48) | 0.31 | 1.23(0.88,1.71) | 0.22 | 1.57(1.00,2.46) | 0.05 |
| Q4 | 1.27(0.94,1.70) | 0.11 | 0.84(0.58,1.21) | 0.34 | 1.02(0.64,1.63) | 0.93 |
| Q5 | 1.41(1.07,1.85) | 0.02 | 0.86(0.60,1.23) | 0.4 | 0.89(0.56,1.42) | 0.62 |

**Table S3. Univariate linear regression analysis between covariates and neutrophil number and WBC count**

| **Characteristics** | **Neutrophil** | | **WBC** | |
| --- | --- | --- | --- | --- |
|  | **OR (95%CI)** | ***P*** | **OR (95%CI)** | ***P*** |
| Age | -0.01(-0.01,0.00) | <0.001 | -0.01(-0.02, -0.01) | <0.0001 |
| Gender |  |  |  |  |
| Female | ref | ref | ref | ref |
| Male | -0.12(-0.23, -0.01) | 0.04 | -0.11(-0.26,0.04) | 0.14 |
| Education level |  |  |  |  |
| Higher than high school | ref | ref | ref | ref |
| High school or equivalent | 0.28(0.16,0.39) | <0.0001 | 0.4(0.23,0.57) | <0.0001 |
| Under high school | 0.25(0.08,0.42) | 0.005 | 0.35(0.14,0.56) | 0.001 |
| Marital status |  |  |  |  |
| Married/cohabiting | ref | ref | ref | ref |
| Divorced/separated/widowed | 0.27(0.13,0.42) | <0.001 | 0.43(0.20,0.66) | <0.001 |
| Never married | 0.13(-0.02,0.29) | 0.09 | 0.26(0.06,0.45) | 0.01 |
| Race/ethnicity |  |  |  |  |
| Mexican-American | ref | ref | ref | ref |
| Non-Hispanic Black | -0.8(-0.95, -0.65) | <0.0001 | -0.87(-1.05, -0.68) | <0.0001 |
| Non-Hispanic White | -0.12(-0.26, 0.01) | 0.07 | -0.24(-0.42, -0.07) | 0.01 |
| Other race | -0.18(-0.35, -0.01) | 0.03 | -0.3(-0.51, -0.10) | 0.004 |
| PIR |  |  |  |  |
| Low (<1.31) | ref | ref | ref | ref |
| Medium (1.31-3.5) | -0.21(-0.35, -0.06) | 0.01 | -0.29(-0.47, -0.12) | 0.002 |
| High (>3.5) | -0.37(-0.50, -0.24) | <0.0001 | -0.56(-0.71, -0.40) | <0.0001 |
| BMI (kg/m2) |  |  |  |  |
| Underweight/normal (<25) | ref | ref | ref | ref |
| Overweight (25-29.9) | 0.19(0.07,0.30) | 0.002 | 0.3(0.14,0.46) | <0.001 |
| Obese (>29.9) | 0.67(0.54,0.80) | <0.0001 | 1.05(0.87,1.23) | <0.0001 |
| Smoke |  |  |  |  |
| No | ref | ref | ref | ref |
| Yes | 0.42(0.32,0.51) | <0.0001 | 0.62(0.50,0.74) | <0.0001 |
| Alcohol use |  |  |  |  |
| No | ref | ref | ref | ref |
| Yes | -0.01(-0.12,0.10) | 0.91 | -0.04(-0.18,0.11) | 0.61 |
| AMED |  |  |  |  |
| Q1 | ref | ref | ref | ref |
| Q2 | -0.14(-0.29, 0.00) | 0.05 | -0.16(-0.34, 0.02) | 0.07 |
| Q3 | -0.24(-0.39, -0.09) | 0.002 | -0.33(-0.52, -0.14) | <0.001 |
| Q4 | -0.45(-0.60, -0.30) | <0.0001 | -0.59(-0.78, -0.40) | <0.0001 |
| Q5 | -0.49(-0.65, -0.32) | <0.0001 | -0.71(-0.92, -0.50) | <0.0001 |
| AHEI |  |  |  |  |
| Q1 | ref | ref | ref | ref |
| Q2 | -0.01(-0.21, 0.19) | 0.91 | -0.08(-0.32, 0.16) | 0.52 |
| Q3 | -0.31(-0.49, -0.12) | 0.001 | -0.42(-0.65, -0.20) | <0.001 |
| Q4 | -0.31(-0.50, -0.12) | 0.001 | -0.5(-0.71, -0.28) | <0.0001 |
| Q5 | -0.56(-0.73, -0.39) | <0.0001 | -0.83(-1.02, -0.63) | <0.0001 |
| DII |  |  |  |  |
| Q1 | ref | ref | ref | ref |
| Q2 | 0.21(0.08,0.34) | 0.002 | 0.33(0.15,0.51) | <0.001 |
| Q3 | 0.21(0.05,0.36) | 0.01 | 0.39(0.18,0.59) | <0.001 |
| Q4 | 0.43(0.26,0.60) | <0.0001 | 0.67(0.46,0.88) | <0.0001 |
| Q5 | 0.3(0.10,0.49) | 0.003 | 0.56(0.31,0.82) | <0.0001 |
| HEI |  |  |  |  |
| Q1 | ref | ref | ref | ref |
| Q2 | 0.04(-0.17, 0.25) | 0.7 | -0.01(-0.27, 0.24) | 0.92 |
| Q3 | -0.14(-0.34, 0.06) | 0.16 | -0.21(-0.47, 0.04) | 0.1 |
| Q4 | -0.34(-0.51, -0.17) | <0.001 | -0.56(-0.78, -0.33) | <0.0001 |
| Q5 | -0.45(-0.62, -0.28) | <0.0001 | -0.75(-0.96, -0.55) | <0.0001 |

**Table S4. Multivariable logistic regression analysis between neutrophil number and WBC count and RA**

| **Characteristics** | **Model 1** | | **Model 2** | | **Model 3** | |
| --- | --- | --- | --- | --- | --- | --- |
|  | **OR (95% CI)** | ***P*** | **OR (95% CI)** | ***P*** | **OR (95% CI)** | ***P*** |
| Neutrophil | 1.17(1.10,1.25) | <0.0001 | 1.11(1.04,1.19) | 0.002 | 1.10(1.03,1.17) | 0.01 |
| WBC | 1.08(1.02,1.14) | 0.01 | 1.05(1.00,1.09) | 0.04 | 1.04(0.99,1.09) | 0.11 |

Notes: Model 1: adjusted for gender, age; Model 2: adjusted for gender, age, BMI, smoking, alcohol consumption; Model 3: Adjusted for age, gender, race/ethnicity, PIR, education level, marital status, BMI, smoking status, alcohol consumption and four dietary patterns

**Table S5. Univariate linear regression analyses between dietary live microbes and neutrophil number and WBC count**

| **Characteristics** | **OR (95%CI)** | ***P*** |
| --- | --- | --- |
| Neutrophil |  |  |
| Low live microbes group | ref | ref |
| Medium live microbes group | -0.14(-0.26, -0.02) | 0.03 |
| High live microbes group | -0.2(-0.34, -0.07) | 0.003 |
| WBC |  |  |
| Low live microbes group | ref | ref |
| Medium live microbes group | -0.22(-0.38, -0.07) | 0.004 |
| High live microbes group | -0.37(-0.56, -0.18) | <0.001 |

**Table S6. Multivariable linear regression analyses between dietary live microbes and neutrophil number and WBC count**

| **Characteristics** | **Model 1** | | **Model 2** | | **Model 3** | |
| --- | --- | --- | --- | --- | --- | --- |
|  | **OR (95% CI)** | ***P*** | **OR (95% CI)** | ***P*** | **OR (95% CI)** | ***P*** |
| Neutrophil |  |  |  |  |  |  |
| Low live microbes group | ref | ref | ref | ref | ref | ref |
| Medium live microbes group | -0.13(-0.25, -0.01) | 0.03 | -0.07(-0.19, 0.05) | 0.24 | -0.02(-0.14, 0.10) | 0.72 |
| High live microbes group | -0.21(-0.34, -0.08) | 0.002 | -0.12(-0.25, 0.01) | 0.07 | -0.09(-0.21, 0.04) | 0.17 |
| WBC |  |  |  |  |  |  |
| Low live microbes group | ref | ref | ref | ref | ref | ref |
| Medium live microbes group | -0.2(-0.36, -0.05) | 0.01 | -0.11(-0.26, 0.04) | 0.14 | -0.02(-0.17, 0.12) | 0.74 |
| High live microbes group | -0.38(-0.57, -0.19) | <0.001 | -0.23(-0.40, -0.06) | 0.01 | -0.15(-0.33, 0.02) | 0.08 |

Notes: Model 1: adjusted for gender, age; Model 2: adjusted for gender, age, BMI, smoking, alcohol consumption; Model 3: Adjusted for age, gender, race/ethnicity, PIR, education level, marital status, BMI, smoking status, alcohol consumption, and four dietary patterns

**Table S7.** **Univariate logistic regression analysis between dietary live microbes and RA and other non-OA arthritis after removing non-dietary prebiotic/probiotic data**

| **Characteristics** | **OR (95%CI)** | ***P*** |
| --- | --- | --- |
| RA |  |  |
| Low live microbes group | ref | ref |
| Medium live microbes group | 0.99(0.80,1.22) | 0.91 |
| High live microbes group | 0.65(0.50,0.84) | 0.001 |
| Other types of arthritis |  |  |
| Low live microbes group | ref | ref |
| Medium live microbes group | 0.96(0.75,1.22) | 0.72 |
| High live microbes group | 0.62(0.45,0.85) | 0.003 |

**Table S8.** **Multivariable logistic regression analysis between dietary live microbes and RA and other non-OA arthritis after removing non-dietary prebiotic/probiotic data**

| **Characteristics** | **Model 1** | | **Model 2** | | **Model 3** | |
| --- | --- | --- | --- | --- | --- | --- |
|  | **OR (95% CI)** | ***P*** | **OR (95% CI)** | ***P*** | **OR (95% CI)** | ***P*** |
| RA |  |  |  |  |  |  |
| Low live microbes group | ref | ref | ref | ref | ref | ref |
| Medium live microbes group | 0.80(0.63,1.02) | 0.07 | 0.89(0.69,1.14) | 0.34 | 1.09(0.84,1.42) | 0.49 |
| High live microbes group | 0.58(0.44,0.76) | <0.001 | 0.67(0.51,0.88) | 0.005 | 0.89(0.67,1.19) | 0.43 |
| Other types of arthritis |  |  |  |  |  |  |
| Low live microbes group | ref | ref | ref | ref | ref | ref |
| Medium live microbes group | 0.78(0.61,1.01) | 0.06 | 0.87(0.68,1.12) | 0.27 | 0.97(0.75,1.25) | 0.82 |
| High live microbes group | 0.55(0.40,0.76) | <0.001 | 0.63(0.45,0.88) | 0.01 | 0.69(0.50,0.97) | 0.03 |

Notes: Model 1: adjusted for gender, age; Model 2: adjusted for gender, age, BMI, smoking, alcohol consumption; Model 3: Adjusted for age, gender, race/ethnicity, PIR, education level, marital status, BMI, smoking status, alcohol consumption, neutrophil number, WBC count and four dietary patterns
